# The Anti-Vascular Endothelial Growth Factor Receptor 1 (VEGFR-1) D16F7 Monoclonal Antibody Inhibits Melanoma Adhesion to Soluble VEGFR-1 and Tissue Invasion in Response to Placenta Growth Factor

**DOI:** 10.3390/cancers14225578

**Published:** 2022-11-14

**Authors:** Maria Grazia Atzori, Claudia Ceci, Federica Ruffini, Manuel Scimeca, Rosella Cicconi, Maurizio Mattei, Pedro Miguel Lacal, Grazia Graziani

**Affiliations:** 1 Department of Systems Medicine, University of Rome Tor Vergata, Via Montpellier 1, 00133 Rome, Italy; 2 Departmental Faculty of Medicine and Surgery, Saint Camillus International University of Health Sciences, Via di Sant’Alessandro, 8, 00131 Rome, Italy; 3 Department of Experimental Medicine, University of Rome Tor Vergata, Via Montpellier 1, 00133 Rome, Italy; 4 Interdepartmental Center for Comparative Medicine, Alternative Techniques and Aquaculture (CIMETA), University of Rome Tor Vergata, Via Montpellier 1, 00133 Rome, Italy; 5 Department of Biology, University of Rome Tor Vergata, Via della Ricerca Scientifica 1, 00133 Rome, Italy; 6 Laboratory of Molecular Oncology, IDI-IRCCS, Via dei Monti di Creta 104, 00167 Rome, Italy

**Keywords:** PlGF, VEGF-A, VEGFR-1, melanoma, metastasis

## Abstract

**Simple Summary:**

Melanoma is an aggressive cancer type with a high tendency to spread to distant body sites, including bones. Despite the availability of effective therapies, many patients still do not fully benefit from treatment. The aim of our study was to evaluate the therapeutic potential of inhibiting the activation of the vascular endothelial growth factor receptor (VEGFR-1) by placenta growth factor (PlGF) using an investigational anti-VEGFR-1 monoclonal antibody (D16F7 mAb). The VEGFR-1 receptor is expressed by endothelial cells of blood vessels that nourish the tumor, protumoral macrophages and melanoma cells. Results indicate that PlGF stimulates the ability of melanoma to infiltrate the surrounding tissues and that treatment with D16F7 mAb counteracts melanoma properties, which contribute to tumor spread, reducing the invasiveness of the tumor and its tropism toward bone tissue. Therefore, blockade of VEGFR-1 stimulation by PlGF represents a suitable strategy to restrain the metastatic potential of melanoma.

**Abstract:**

Placenta growth factor (PlGF) is a member of the vascular endothelial growth factor (VEGF) family involved in tumor-associated angiogenesis and melanoma invasion of the extra-cellular matrix (ECM) through activation of membrane VEGF receptor 1 (VEGFR-1). A soluble VEGFR-1 (sVEGFR-1) form is released in the ECM, where it sequesters proangiogenic factors and stimulates endothelial or tumor cell adhesion and chemotaxis through interaction with α5β1 integrin. The anti-VEGFR-1 monoclonal antibody (D16F7 mAb) inhibits VEGF-A or PlGF-mediated signal transduction without affecting ligand interaction, thus preserving sVEGFR-1 decoy function. The aim of this study was to investigate whether D16F7 mAb hampers melanoma spread by in vitro analysis of cell adhesion to sVEGFR-1, ECM invasion, transmigration through an endothelial cell monolayer and in vivo evaluation of tumor infiltrative potential in a syngeneic murine model. Results indicate that D16F7 mAb significantly inhibits melanoma adhesion to sVEGFR-1 and ECM invasion, as well as transmigration in response to PlGF. Moreover, treatment of melanoma-bearing mice with the anti-VEGFR-1 mAb not only inhibits tumor growth but also induces a significant reduction in bone infiltration associated with a decrease in PlGF-positive melanoma cells. Furthermore, D16F7 mAb reduces PlGF production by melanoma cells. Therefore, blockade of PLGF/VEGFR-1 signaling represents a suitable strategy to counteract the metastatic potential of melanoma.

## 1. Introduction

Placenta growth factor (PlGF) is a member of the vascular endothelial growth factor family, which, in contrast to vascular endothelial growth factor-A (VEGF-A), interacts with the VEGF receptor 1 (VEGFR-1) but not with the type 2 receptor (VEGFR-2) [1]. Besides human placenta, from which it was initially isolated [2], a number of cells and tissue types can produce PlGF, including heart, lung, liver, thyroid, skeletal muscle, bone and the tumor itself [3]. Of the four human PlGF isoforms produced, PlGF-1 and PlGF-2 are the most abundant. Murine PlGF is highly similar to human PlGF-2 and, like the latter, contains a heparin-binding domain [4]. PlGF is not involved in normal physiological processes, whereas it is expressed under pathological conditions, including cancer [1]. Within the tumor mass, the growth factor can activate VEGFR-1 expressed on endothelial cells, pericytes and smooth muscle cells, directly stimulating neovessel formation [5]. It also exerts proangiogenic effects by acting on nonvascular cells, promoting the recruitment of myeloid progenitors and polarization of M2 macrophages, which release VEGF-A, as well as other angiogenic factors, such as transforming growth factor-β1 [5,6,7,8]. Accordingly, the demonstrated antitumor and antimetastatic activity of host-produced histidine-rich glycoprotein seems to involve downregulation of PlGF in tumor-associated macrophages, which results in skewing of their polarization from the M2 to the antitumor M1 phenotype [9]. Furthermore, PlGF can directly stimulate migration through the extracellular matrix (ECM) of VEGFR-1-positive melanoma cells, favoring the metastatic process [6]. After binding to VEGFR-1, PlGF is able to stimulate the survival, proliferation, migration and activation of the target cell. Receptor activation is followed by phosphorylation of specific tyrosine residues of the kinase receptor domain, which are distinct from those phosphorylated upon VEGF-A binding [10].

Activation of VEGFR-1 has been shown to be involved in epithelial–mesenchymal transition (EMT), which confers on cancer cells a more invasive phenotype, and to promote metalloprotease activation [11]. Interestingly, PlGF is frequently expressed by melanoma cell lines derived from metastatic lesions compared to those obtained from primary tumors [12]. Thus, PlGF secretion seems to increase during melanoma progression. Furthermore, syngeneic melanoma cells injected in transgenic mice overexpressing PlGF in the skin showed a higher growth rate and number of distant metastases compared to control mice [13]. In the case of intrahepatic cholangiocarcinoma, PlGF blockade induced a reduction in metastatic dissemination, enhanced chemosensitivity and increased survival of tumor-bearing mice [14]. Moreover, high PlGF serum levels predicted a lack of benefit from the anti-VEGF-A mAb bevacizumab [15,16].

Our previous data indicated that the anti-VEGFR-1 D16F7 mAb generated in our laboratories is able to inhibit melanoma and glioblastoma cell chemotaxis in response to PlGF and VEGF-A and that this activity translates to efficient antitumor activity in preclinical in vivo models, even when mAb is administered as a single agent [17,18,19,20]. The D16F7 mAb is characterized by a novel mechanism of action, as it inhibits VEGF-A- or PlGF-mediated signal transduction without hampering ligand binding to the extracellular receptor domain [17]. The positive implication of this particular mechanism relies on the ability of mAb to leave intact the antiangiogenic activity of the VEGFR-1 soluble form (sVEGFR-1), which is released by endothelial and tumor cells in the ECM, where it sequesters receptor ligands (decoy receptor function) [6]. The sVEGFR-1 comprises the growth factor binding domain of the membrane receptor, and following its interaction with VEGF-A and PlGF, it prevents the stimulation of the transmembrane tyrosine kinase [21]. An additional function of the sVEGFR-1 is that, as a component of the ECM, it can stimulate endothelial and tumor cell adhesion and migration through a direct interaction with α5β1 integrin, which results in the generation of a motile phenotype [22,23,24]. High expression of sVEGFR-1 has been found to play a role in melanoma progression, promoting ECM invasion by melanoma cells and metastasis localization [25].

In the present study, we investigated whether the anti-VEGFR-1 D16F7 mAb may hamper the ability of melanoma cells to adhere to the sVEGFR-1, invade the ECM and transmigrate through an endothelial cell monolayer in response to PlGF and whether these effects may translate in vivo into a reduced invasiveness and infiltrative potential of melanoma in the surrounding tissues.

## 2. Materials and Methods

### 2.1. Cell Lines and Culture Conditions

The immortalized human endothelial cell line HUV-ST was generated in our laboratory [26] and maintained in culture in endothelial growth factor medium (EGM-2; Cat No. CC-3156, Lonza, Basel, Switzerland) supplemented with 0.4 mg/mL geneticin and 5 μg/mL puromycin at 37 °C in a 5% CO_2_ humidified atmosphere. HUV-ST cells were used at passages between 33 and 40. Murine B16F10, human WM115 and WM266-4 melanoma cell lines were purchased from the American Type Culture Collection (Cat No. CRL-6475, CRL-1675 and CRL-1676, ATCC, Manassas, VA, USA) and cultured in RPMI 1640 medium (Cat No. R0883, Sigma-Aldrich, St. Louis, MO, USA) supplemented with 10% fetal bovine serum (FBS) (Cat No. F7524, Sigma-Aldrich, St. Louis, MO, USA). All the cell lines tested in this study were previously characterized for the expression of VEGFR-1 [17,25,26].

### 2.2. Cell Adhesion Assay

Adhesion to sVEGFR-1 was evaluated as previously described [22,25]. Solid support for adhesion assay was prepared by incubating 96-well plates overnight at room temperature with 50 µL of 5 µg/mL recombinant human sVEGFR-1 (Cat No. S01-010, Reliatech, Wolfenbüttel, Germany) (for HUV-ST, WM266-4 cells), recombinant murine sVEGFR-1D7/Fc chimera (Cat No. SFC-M05, Reliatech) (for B16F10 cells) or, as control, fibronectin (Cat No. F0895, Merck, Darmstadt, Germany) solubilized in PBS. Thereafter, the coating solution was removed, and the well surface was blocked with 100 µL of 1% fatty-acid-free bovine serum albumin (BSA; Cat No. 10775835001, Roche, Basel, Switzerland) in PBS for 2 h. Before adding the cells, plates were treated with 50 µL of the indicated concentrations of the anti-VEGFR-1 D16F7 mAb [17,18,19,20] for 30 min. Large-scale production and purification of D16F7 mAb for in vitro and in vivo experiments was performed by Cell Essentials Inc. (Boston, MA, USA) from a hybridoma generated in our laboratories [17]. Then, 5 × 10^4^ cells per well were plated in 50 µL of serum-free medium containing 0.1% BSA. After incubation at 37 °C for 1–2 h (as indicated in the legend of each figure depending on the cell line tested), wells were washed with PBS, and attached cells were fixed with ethanol and stained with 0.5% crystal violet (Cat No. C0775, Sigma-Aldrich). Attachment efficiency was determined by dissolving the dye in 100 µL of 10% acetic acid, and spectrophotometric measurement of the absorbance was conducted at 595 nm using a microplate reader (Infinite F50, Tecan, Männedorf, Switzerland).

### 2.3. Transendothelial Cell Migration Assay

In vitro transendothelial migration was performed as described in [27], with minor modifications, using Boyden chambers equipped with 8 μm pore diameter polycarbonate filters (Cat No. 150446, Nuclepore Whatman^TM^, Cytiva, Marlborough, MA, USA) coated with 5 μg/mL gelatin (Cat No. G9391, Sigma-Aldrich). HUV-ST cells were plated in the upper compartment of the Boyden chamber (2.5 × 10^5^ cells/chamber) in 200 µL of EGM2 complete medium. After 3 h, cells were treated with 100 ng/mL TNF-α (Cat No. 11343015, Immunotools, Friesoythe, Germany) in the same volume of endothelial basal medium (EBM-2; Lonza) plus 0.1% FBS. WM115 cells were labeled with 1 µM calcein-AM (Cat No. C1430, Invitrogen™, Waltham, MA, USA) in serum-free medium (2 × 10^6^ cells/mL) for 30 min at 37 °C and suspended at 10^6^ cells/mL in serum-free medium containing 0.5% BSA. To test the effect of D16F7 mAb, melanoma cells were preincubated with 5 µg/mL of D16F7 mAb or control antibody (mouse IgG1, Cat No. MAB002, R&D Systems, Biotechne, Minneapolis, MN, USA) in a rotating wheel for 30 min at room temperature. Cells (2 × 10^5^/200 µL) were then loaded in new Boyden chambers containing the filters with the activated endothelium monolayer, and a transmigration assay was performed in the presence of either D16F7 or control antibodies. The lower chamber was previously filled with medium plus 0.5% BSA in the absence or presence of 50 ng/mL recombinant human PlGF (Cat No. 300-016, Reliatech). After 5 h, filters were fixed with 3.7% paraformaldehyde, and the remaining cells on the upper surface were removed using a cotton swab. Membranes were assembled on a microscope slide, and photographs of the green fluorescent cells were taken at 100× magnification with an Eclipse TE2000-S microscope and NIS-Elements software (Nikon, Minato, Japan). Independent fields for each well were analyzed, and transmigration was quantified using the ImageJ software (http://rsb.info.nih.gov/ij); National Institutes of Health, Bethesda, MD, USA).

### 2.4. Invasion Assay

B16F10 cell invasiveness was evaluated using Boyden chambers equipped with 8 μm pore diameter polycarbonate filters (Nuclepore Whatman^TM^) coated with 20 μg of Matrigel (Cat No. 3432-005-01, R&D Systems) [28]. Cells were suspended in invasion medium (0.1% BSA in RPMI 1640 supplemented with 1 μg/mL heparin, Cat No. H3149, Sigma-Aldrich) and loaded (5 × 10^4^ cells/200 µL) into the upper compartment of the chambers. Invasion medium with or without recombinant murine PlGF (50 ng/mL; Cat No. M30-019, Reliatech) was added to the lower compartment of the chambers. After incubation at 37 °C in a CO_2_ incubator for 2 h, filters were removed from the chambers, and cells were fixed in ethanol for 5 min and stained with 0.5% crystal violet for 15 min. Cells attached to the upper side of the filters were removed by wiping them with a cotton swab, and invaded cells attached to the lower surface of the filters were counted under the microscope (200× magnification).

### 2.5. Quantification of PlGF-2 in B16F10 Cell Culture Conditioned Media by ELISA

B16F10 cells (2.5 × 10^5^/well in 2 mL of complete medium) were seeded in 6-well plates and allowed to grow for 48 h in the absence or presence of D16F7 mAb (10 μg/mL). Conditioned media from B16F10 cells were obtained by incubating cultures for an additional 24 h, always in the presence or absence of the antibody, in 2 mL of 0.1% BSA/RPMI 1640 medium. These culture conditions did not significantly affect cell viability (Supplementary Figure S1). Supernatants were concentrated at least 5-fold in Vivaspin concentrators (Cat No. VS04T02, Sartorius, Göttingen, Germany). Cells were detached from the flasks with PBS/EDTA and counted. Detection of growth factor levels was performed using a DuoSet^®^ mouse PlGF-2 kit (Cat No. DY465, R&D Systems) following the manufacturer’s instructions [29]. Optical density was measured at 450 nm (reference wavelength, 540 nm) in a microplate reader (Infinite F50, Tecan). Levels of PlGF-2 in cell-conditioned medium were normalized according to the total number of cells.

### 2.6. Immunoblot Analysis

The murine sVEGFR-1D7/Fc chimera was run in 8% SDS-polyacrylamide gels (Cat No. A3574; Sigma-Aldrich) and transferred to supported nitrocellulose membranes (Cat No. 10600002; Amersham™ Protran, Euroclone, Pero, Milan, Italy) by standard techniques [17]. Membranes were incubated with the D16F7 mAb diluted at 1 µg/mL, and immunodetection was performed using anti-mouse Ig/horseradish peroxidase secondary antibody (1:1000; Cat No. A4416, Merck) and the Clarity Western ECL substrate from Bio-Rad Laboratories (Cat No. 1705061; Hercules, CA, USA). The molecular weight marker used was from Euroclone (Cat No. EPS025500).

### 2.7. In Vivo Studies

To investigate the effect of the anti-D16F7 mAb on in vivo melanoma growth, B16F10 cells (10^5^) were injected intramuscularly (i.m.) in the left hind limb of 6-week-old male BDF1 inbred mice (24–26 g weight, Cat No. 099BDF-1; Charles River, Willmington, MA, USA). Mice were kept in a temperature-controlled room (25 ± 2 °C) at 50% relative humidity with a 12/12 h light/dark cycle and had ad libitum access to food and water. When tumors were palpable, animals were randomized and treated intraperitoneally (i.p.) with vehicle (PBS) or the anti-VEGFR-1 D16F7 mAb (10 mg/kg every other day for six administrations), as previously described [17,18,19,20].

The sample size (six mice for each experimental group) was calculated based on our previous studies [17,20]. Because cells were inoculated i.m. in the leg of the animals, only two dimensions could be measured: length and width. Therefore, tumor growth was monitored by measuring tumor mass in two dimensions with a digital caliper, and melanoma volumes were calculated according to the following formula: tumor volume (mm^3^) = [length (mm) × width^2^ (mm^2^)]/2.

Animals were sacrificed on day 12, when tumor volume in the control mice reached half of the maximal acceptable size (i.e., 1 cm^3^). After the sacrifice, samples were fixed in 4% paraformaldehyde for 24 h and paraffin-embedded. Serial sections measuring 3 µm (three for each sample) were stained with hematoxylin–eosin for morphological analysis. Specifically, hematoxylin–eosin sections were used to evaluate the infiltration of cancer cells in both muscle and bone tissues.

For immunohistochemistry, 4 µm paraffin serial sections (three for each sample) were used to evaluate the expression of PlGF in tumor cells. Briefly, deparaffinized sections were treated with EDTA citrate (pH 7.8) at 95 °C for 30 min for antigen retrieval. Then, sections were incubated with a rabbit polyclonal anti-mouse PlGF antibody for 30 min (1:100; ab196666, lot GR255736-57, Abcam, Cambridge, UK) and HRP-goat anti-rabbit secondary antibody (Cat No. NB7160, Novus Biological, Littleton, CO, USA). Reactions were revealed using an HRP-DAB detection kit (UCS Diagnostic, Rome, Italy). Immunoreaction was evaluated as a percentage of PlGF-positive tumor cells/high-power field.

To further confirm immunohistochemical analysis by discriminating PlGF-positive cancer cells from the endothelial cells, dual-color immunofluorescence investigations were performed. To this end, immunofluorescence analysis of PlGF expression (Texas red) was performed simultaneously with a marker for melanoma cells (HMB45-FITC). After antigen retrieval, autofluorescence was reduced by tetrahydroborate solution for 40 min. Slides were incubated with the anti-PlGF antibody (1:100; ab196666, Abcam) and the anti-HMB 45 antibody (1:100; HMB45 clone, Cat No. 790-4366, Ventana, Roche) for 30 min. Washes were performed with PBS/Tween 20 pH 7.6. Reactions were revealed using FITC-rabbit anti-mouse secondary antibodies (Cat No. NB7543, Novus Biologicals) for HMB45 and Texas red-goat anti-rabbit secondary antibodies (Cat No. NB120-6719, Novus Biologicals) for PlGF. Lastly, DAPI (1 µg/mL; Cat No. NBP2-31156, Novus Biologicals) was used to stain the nucleus.

All procedures involving mice and their care were performed in compliance with our institutional animal care guidelines and following national and international directives (D.L. 4 March 2014, No. 26; directive 2010/63/EU of the European Parliament and Council). The experimental protocol was approved by the Animal Care and Use Committee at the institution involved in this study and by the Italian Ministry of Health (authorization N. 527/2019-PR; see also, Institutional Review Board Statement).

### 2.8. Statistical Analysis

All statistical analyses were performed using GraphPad Prims 6 software for Windows (San Diego, CA, USA). Statistical analysis of the differences between two groups was performed using the unpaired Student’s *t* test if data passed the normality test (omnibus K2 D’Agostino–Pearson test) [30,31] or, in the case of non-normally distributed data, by the Mann–Whitney U test. Omnibus K2 D’Agostino–Pearson is the test recommended by GraphPad Prims 6 for evaluation of normality, as it is a versatile and powerful test. It calculates skewness and kurtosis to evaluate the extent of data distribution from Gaussian in terms of asymmetry and shape. Thereafter, the test calculates the extent to which each value differs from the expected value with a Gaussian distribution, determining a single *p* value from the sum of these discrepancies. For multiple comparisons, the Kruskal–Wallis test was used, followed by Dunn’s post hoc analysis. *p* values < 0.05 were considered statistically significant.

## 3. Results

### 3.1. Involvement of VEGFR-1 in Melanoma Cell Invasiveness and Adhesion to ECM

**Figure 1:**
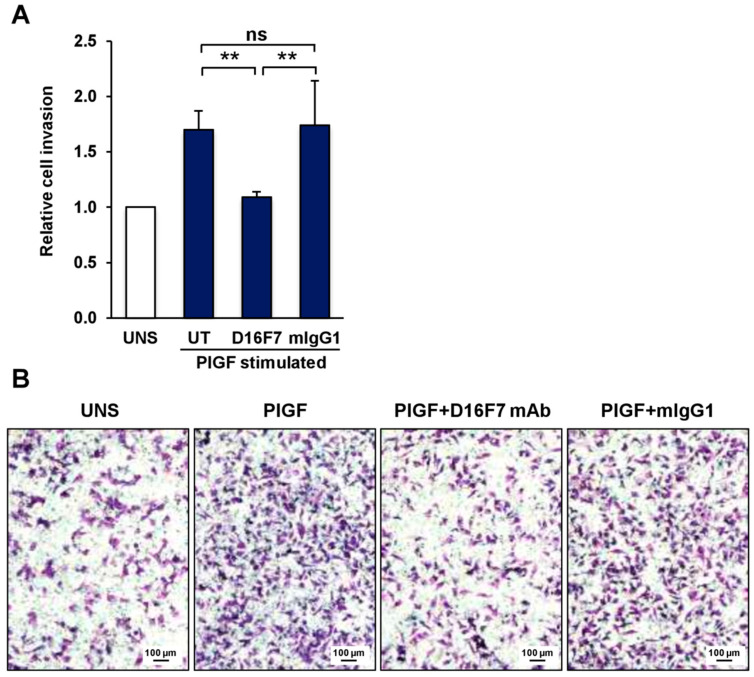
The anti-VEGFR-1 D16F7 mAb inhibits the ability of murine melanoma cells to invade the ECM in response to PlGF. (**A**) The ability of B16F10 cells to invade the ECM upon murine PlGF stimulation (50 ng/mL) in the absence (UT, untreated cells) or presence of 5 μg/mL of D16F7 mAb or control mouse IgG mAb (mIgG1) was analyzed in Boyden chambers equipped with Matrigel-coated filters (5 × 10^4^ cells/chamber, 2 h incubation). The number of invading cells was counted as indicated in the Materials and Methods section, and the results for PlGF-stimulated cells were expressed as relative invasion with respect to the control, unstimulated cells (UNS). The histogram represents the arithmetic mean values ± SD of 5–7 chambers for each experimental condition from three independent experiments. Statistical analysis was performed by Kruskal–Wallis test, followed by Dunn’s post hoc analysis: **, *p* < 0.01; ns, not significant. (**B**) Representative photographs of invaded cells are shown (100× magnification). Scale bars represent 100 µm.

The previously observed ability of D16F7 mAb to reduce melanoma growth in mice models was accompanied by a reduced infiltration of the tissues surrounding the tumor. This finding can be explained by the antibody inhibitory effects on neovessel formation and on the activity of tumor-associated M2 macrophages [17,20]. In this context, we further investigated the role played by PlGF/VEGFR-1 signaling directly in melanoma cells and, in particular, on their invasive potential, which may contribute to tumor spread. First, we confirmed that D16F7 mAb binds to the murine form of sVEGFR-1 by Western blot analysis (Supplementary Figure S2). Thereafter, the invasiveness of mouse melanoma cells expressing VEGFR-1 (B16F10) was evaluated in an in vitro invasion assay on Matrigel. The results indicate that PlGF significantly stimulated ECM invasion (Figure 1A). Treatment with D16F7 mAb completely abrogated the increased melanoma invasiveness induced by PlGF, whereas a control antibody of the same isotype did not have any effect (Figure 1A). Representative photographs of these results are shown in Figure 1B.

**Figure 2:**
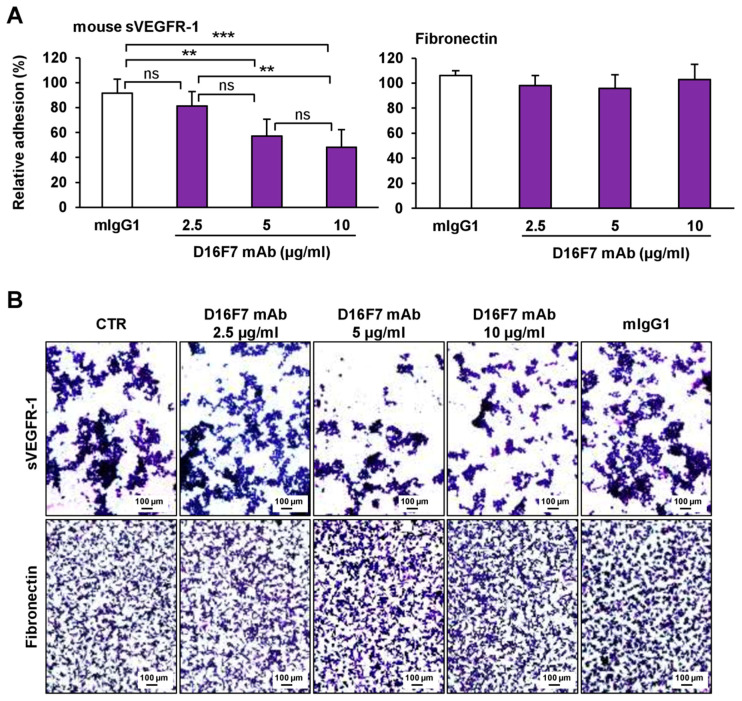
D16F7 mAb hampers the adhesion of B16F10 melanoma cells to murine sVEGFR-1. (**A**) Melanoma cell adhesion was evaluated in 96-well plates coated with mouse VEGFR-1 extracellular domain (sVEGFR-1D7/Fc chimera) or with fibronectin as a control for antibody specificity. BSA was used to determine non-specific adhesion. Cell adhesion to sVEGFR-1 and fibronectin was evaluated in the absence or presence of the indicated concentrations of D16F7 mAb or 10 µg/mL mouse IgG (mIgG1) after 2 h incubation. Each value represents the mean ± SD of 8–9 total determinations from three independent experiments. Adhesion of melanoma cells to sVEGFR-1- or fibronectin-coated wells, after subtraction of background values (non-specific adhesion to BSA-coated wells), was expressed as percent adhesion compared to cells incubated without any antibody. Statistical analysis of the differences between all the groups was performed by Kruskal–Wallis test, followed by Dunn’s post hoc analysis. For cell adhesion to sVEGFR-1, significance symbols were as follows: **, *p* < 0.01; ***, *p* < 0.001; ns, not significant. In the case of cell adhesion to fibronectin, no statistically significant differences were observed between groups. (**B**) Representative photographs are shown of cells adhered on sVEGFR-1 or fibronectin-coated wells for each experimental condition (100× magnification). The scale bars represent 100 µm.

Because the sVEGFR-1 is able to stimulate tumor cell adhesion and migration through a direct interaction with α5β1 integrin, we investigated the influence of D16F7 mAb treatment on sVEGFR-1 activity. To this end, the adhesion of B16F10 melanoma cells to 96-well plates coated with recombinant murine sVEGFR-1 was tested in the absence and presence of D16F7 mAb or of a control murine IgG. The D16F7 antibody was found to reduce, specifically and in a concentration-dependent fashion, the ability of B16F10 cells to adhere to sVEGFR-1 (Figure 2A), which is known to be a component of the ECM produced by endothelial and tumor cells [22]. Conversely, D16F7 mAb did not affect B16F10 melanoma cell adhesion to a VEGFR-1-unrelated substrate, such as fibronectin (Figure 2A). Representative photographs of cell adhesion to sVEGFR-1 or to fibronectin are shown in Figure 2B.

Thus, the anti-VEGFR-1 mAb was able to reduce the invasive response of melanoma cells to PlGF, as well their adhesion to sVEGFR-1, both mechanisms that contribute to trigger the spread of B16F10 cells through the tumor microenvironment.

### 3.2. The Role of PlGF in an In Vivo Murine Melanoma Model

**Figure 3:**
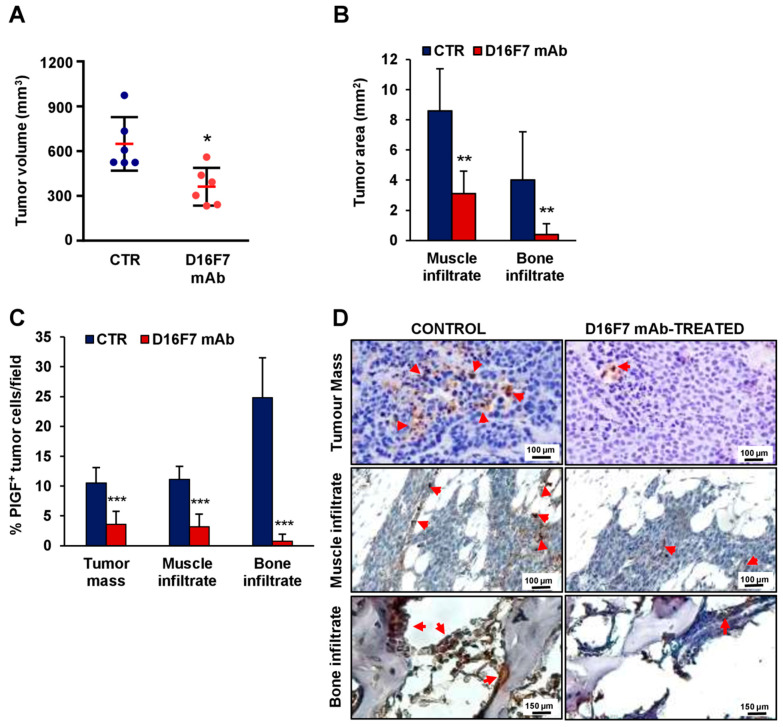
Inhibition of in vivo melanoma growth by the anti-VEGFR-1 D16F7 mAb is associated with a decrease in tissue infiltration by PlGF-positive tumor cells. (**A**) In vivo inhibition of B16F10 melanoma growth by D16F7 mAb. Mice were treated i.p. with the mAb (10 mg/kg) or with PBS as mAb vehicle (CTR), and tumor size (mm^3^) was measured as indicated in the Materials and Methods section. The plot shows differences in melanoma size between control and treated mice on day 12 after tumor challenge (i.e., when the tumor size in control mice reached half of the admissible size). Red horizontal lines indicate the arithmetic mean of tumor size ± SD (*n* = 6). Statistical analysis was performed by Mann–Whitney U test: *, *p* < 0.05. (**B**–**D**) On day 12, three animals were sacrificed for each experimental condition, and tumors were excised and processed for immunohistochemical analysis. (**B**) Morphometric evaluation of muscle and bone tumor infiltration in control and D16F7 mAb-treated mice (7 microscopic fields/group). Statistical analysis of the differences between D16F7 mAb-treated and control samples was performed by Student’s *t*-test (normal distribution): **, *p* < 0.01. (**C**) Analysis of the percentage of PlGF-positive B16F10 melanoma cells in control and D16F7 mAb-treated tumors and surrounding tissues. Sections from three tumor specimens/group were stained for murine PlGF, and the percentage of B16F10 cells positive for PlGF present in the tumor mass and in the tumor infiltrating the bone or the skeletal muscle was calculated. The histogram represents the mean of 14 microscopic fields for each experimental group ± SD. Statistical analysis of the differences between D16F7 mAb-treated and control samples was performed by Student’s *t*-test (normal distribution): ***, *p* < 0.001. (**D**) Representative photographs showing PlGF staining (in tumor mass and muscle infiltrate photographs, the scale bars represent 100 µm; in bone infiltrate photographs, the scale bars represent 150 µm). Arrows point to positively stained cells.

Based on the above-described findings, we investigated the role of the selective VEGFR-1 ligand PlGF in the in vivo invasiveness of B16F10 melanoma cells. In agreement with the results of our previous studies [17,20], in vivo treatment with D16F7 mAb at a dose of 10 mg/Kg in B6D2F1 mice injected i.m. with syngeneic B16F10 melanoma cells induced a reduction in tumor growth of about 50% (Figure 3A). A significant decrease in tumor infiltration was observed in the skeletal muscle and, more markedly, in the bone (Figure 3B). The involvement of PlGF in melanoma infiltration of the surrounding tissues was evaluated by histological determination of the percentage of cells positive for PlGF expression in the tumor mass and in the nearby skeletal muscle and bone. The effect of D16F7 mAb treatment was associated with a strong decrease in the number of PlGF-positive cells, mostly in the bone infiltrate, where the percent decrease of neoplastic PlGF-positive cells was 97% compared to untreated control, whereas in the tumor mass and muscle tissue, the percent reduction was 66 and 71, respectively (Figure 3C,D). Dual-color immunofluorescence investigations analyzing PlGF expression simultaneously with a marker of melanoma cells (HMB45-FITC) showed that the percentage of PlGF and HMB45 co-positive cells was consistent with the immunohistochemical data (Supplementary Figure S3).

These results suggest the possibility that the anti-VEGFR-1 mAb had a direct effect on PlGF production by B16F10 cells or that PlGF-positive cells might be more sensitive to a cytotoxic effect of the antibody treatment. The latter hypothesis was not supported by our previous studies, showing that D16F7 mAb hampers tumor growth by inhibiting tumor cell migration, invasion and vasculogenic mimicry but is devoid of direct cytotoxic effects ([17] and Supplementary Figure S1). Therefore, we tested the first hypothesis by measuring whether the anti-VEGFR-1 mAb might modulate PlGF release by melanoma cells. To this end, B16F10 cells were kept in culture for three days in the presence or absence of the antibody, and PlGF levels secreted in the culture supernatant during the last 24 h were measured (Table 1). Interestingly, an almost 40% reduction in PlGF production was observed in D16F7 mAb-treated B16F10 cells compared to untreated cells (38.8 ± 4.9%).

### 3.3. Effects of D16F7 mAb on Human Melanoma and Endothelial Cell Adhesion to sVEGFR-1

**Figure 4:**
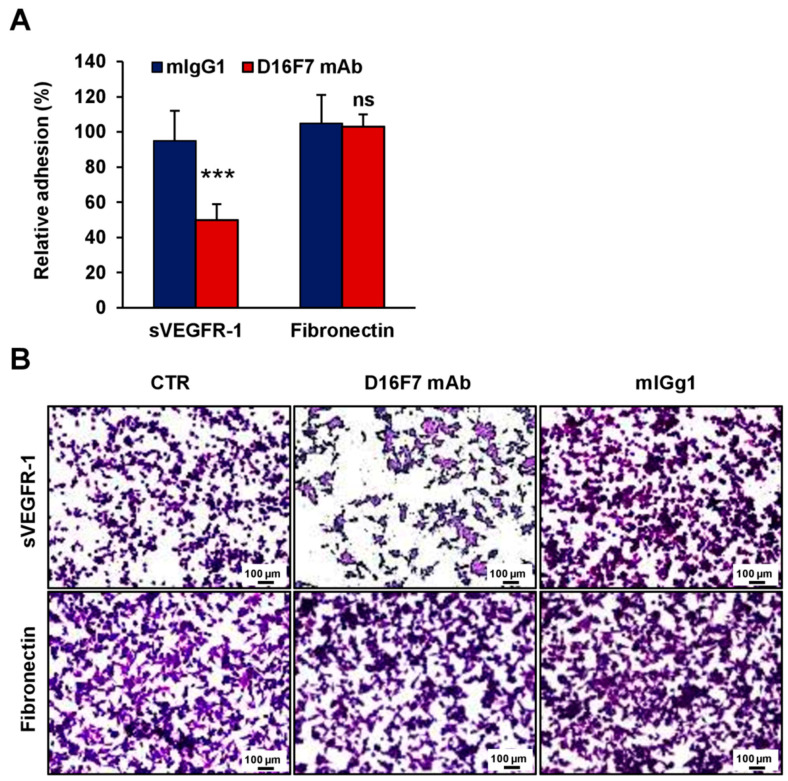
D16F7 mAb inhibits the adhesion of human melanoma cells to sVEGFR-1. (**A**) Adhesion of human melanoma WM266-4 cells was evaluated in 96-well plates coated with recombinant human sVEGFR-1 or with fibronectin as a control for antibody specificity. BSA was used to determine non-specific adhesion. Cell adhesion to sVEGFR-1 and fibronectin was evaluated in the absence or presence of 5 µg/mL D16F7 mAb or mouse IgG (mIgG1) after 1 h incubation. Each value represents the mean ± SD of 12 (sVEGFR-1) or 10 (fibronectin) total determinations from four independent experiments. Adhesion of melanoma cells to sVEGFR-1- or fibronectin-coated wells, after subtraction of background values (nonspecific adhesion to BSA-coated wells), was expressed as percent adhesion compared to cells incubated in the absence of any antibody. Statistical analysis (normal distribution) was performed by Student’s *t*-test, comparing adhesion of D16F7 mAb with mIgG1-treated groups to the sVEGFR-1 substrate: ***, *p* < 0.001. For adhesion to fibronectin, differences between the D16F7 mAb- and mIgG1-treated groups were not statistically significant (ns). (**B**) Representative photographs are shown for each experimental condition (100× magnification). The scale bars represent 100 µm. CTR, control (i.e., untreated cells).

**Figure 5:**
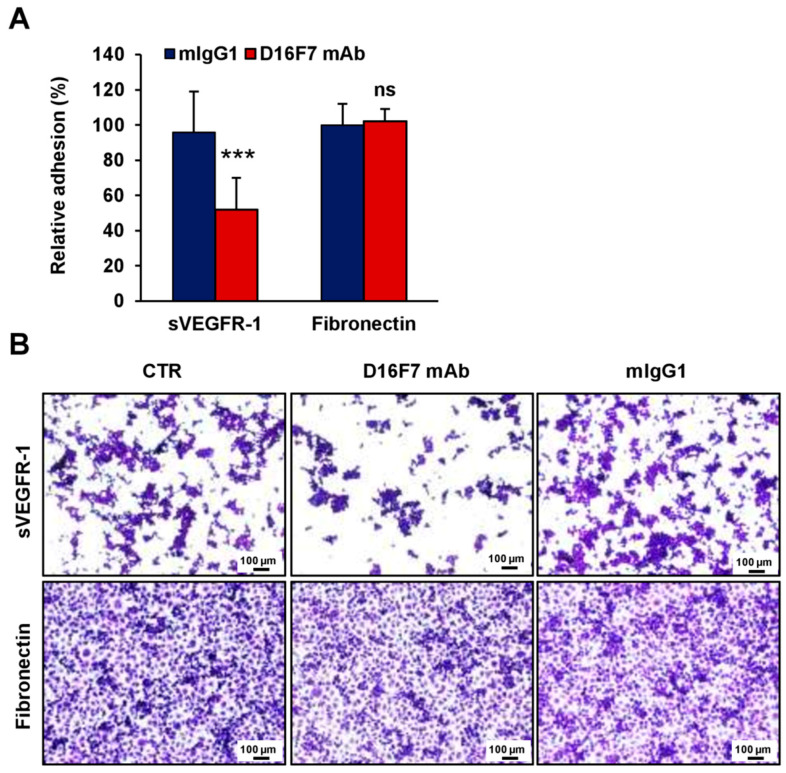
D16F7 mAb hampers the adhesion of human endothelial cells to sVEGFR-1. (**A**) Adhesion of human endothelial cells was evaluated in 96-well plates coated with recombinant human VEGFR-1 or with fibronectin as a control for antibody specificity. BSA was used to determine non-specific adhesion. Cell adhesion to sVEGFR-1 and fibronectin was evaluated in the absence or presence of 5 µg/mL D16F7 mAb or mouse IgG (mIgG1) after 2 h incubation. Each value represents the mean ± SD of 9 (sVEGFR-1) or 7 (fibronectin) total determinations from three independent experiments. Adhesion of melanoma cells to sVEGFR-1- or fibronectin-coated wells, after subtraction of background values (nonspecific adhesion to BSA-coated wells), was expressed as percent adhesion compared to cells incubated in the absence of any antibody. Statistical analysis of the differences between D16F7 mAb- and mIgG1-treated cells was performed by Student’s *t*-test in the case of adhesion to sVEGFR-1 (normal distribution; ***, *p* < 0.001) and by Mann–Whitney U test in the case of adhesion to fibronectin (non-normal distribution; ns, non-significant difference). (**B**) Representative photographs are shown for each experimental condition (100× magnification). The scale bars represent 100 µm. CTR, control (i.e., untreated cells).

In vitro data obtained in the murine model were then confirmed in human models of melanoma and endothelial cells. Human melanoma WM266-4 cells and endothelial HUV-ST cells, known to adhere to sVEGFR-1 [25], were tested for their ability to bind human sVEGFR-1 in the presence or absence of D16F7 mAb (Figure 4 and Figure 5, respectively). The results demonstrated that the antibody markedly reduced the adhesion of these cells to sVEGFR-1-containing ECM, whereas it did not affect melanoma adhesion to fibronectin (Figure 4A for WM266-4 and Figure 5A for HUV-ST cells). On the other hand, a control murine IgG did not have any effect (Figure 4A and Figure 5A). The inhibitory effect was more pronounced compared to that observed with the same mAb concentration in murine B16F10 cells (Figure 2), likely due to the increased ability of D16F7 mAb to interact with the human receptor. In fact, although D16F7 mAb recognizes both the human and murine receptors ([17] and Supplementary Figure S2), the antibody has been produced by immunizing mice with a human VEGFR-1 peptide [17]. Representative photographs of the adhesion results for WM266-4 melanoma cells and for HUV-ST endothelial cells are shown in Figure 4B and Figure 5B, respectively.

**Figure 6:**
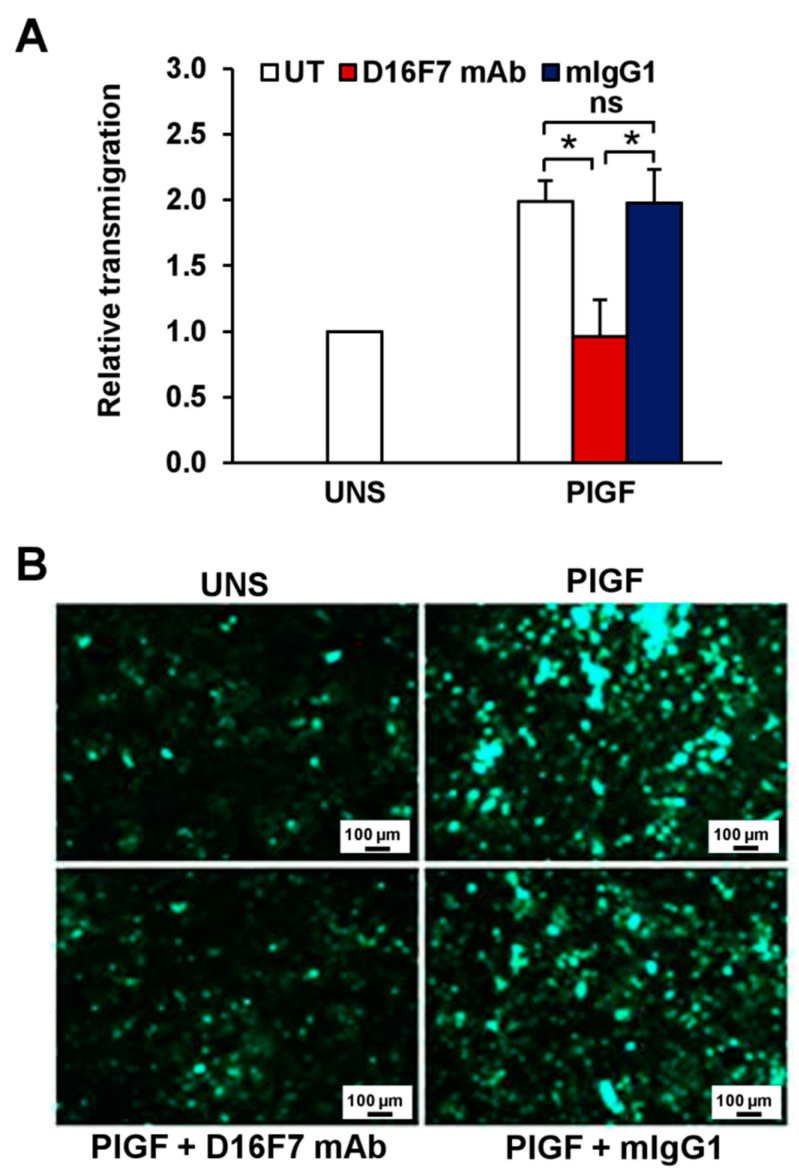
Inhibitory effect of D16F7 mAb on transmigration of human melanoma cells across an activated endothelial cell monolayer in response to human PlGF. (**A**) Monolayers of human endothelial cells (HUV-ST cells) were seeded on the upper side of Boyden chambers and activated with TNF-α. Human melanoma WM115 cells were labelled with calcein-AM and incubated for 30 min at room temperature in the absence (UT) or presence of 5 μg/mL D16F7 mAb or mouse IgG as a control (mIgG1). WM115 cells (2 × 10^5^ cells/chamber) were then loaded on the HUV-ST-activated monolayer and allowed to migrate for 5 h against PlGF (50 ng/mL), which was added as stimulus in the lower compartment of the Boyden chamber. Fluorescence of migrated cells was measured in four to eight fields/filter, and results were expressed as relative transmigration compared to unstimulated cells (UNS). Values in the histogram represent the mean ± SD of five Boyden chambers from three independent experiments. Statistical analysis was performed by Kruskal–Wallis test, followed by Dunn’s post hoc analysis: *, *p* < 0.05; ns, not significant. (**B**) Representative photographs of fluorescent WM115 cells migrated to the lower side of the filter through the endothelial monolayer are shown for each experimental condition (100× magnification). The scale bars represent 100 µm.

Finally, we tested the influence of the anti-VEGFR-1 mAb to hamper melanoma cell transmigration across a human endothelium monolayer, a process involved in tumor progression and metastasis. To this end, the highly invasive human melanoma WM115 cell line originated from a primitive tumor and characterized by high levels of sVEGFR-1 expression [25] was tested. Exposure to PlGF further stimulated WM115 cell transmigration, and the anti-VEGFR-1 mAb completely abrogated migration of melanoma cells through the endothelial monolayer, whereas a murine control IgG did not exert a significant effect (Figure 6A). Representative photographs of transmigrated human melanoma cells are shown in Figure 6B.

## 4. Discussion

In the present study, we demonstrated, for the first time, that PlGF is directly involved in the invasive and infiltrative behavior of malignant melanoma and that blockade of the activation of its cognate receptor, VEGFR-1, by D16F7 mAb counteracts melanoma properties that contribute to tumor spread by inhibiting cell adhesion to ECM components and transmigration through an endothelial monolayer.

Besides being directly required for the angiogenic switch in the growing tumor, PlGF indirectly promotes angiogenesis and lymphoangiogenesis by recruiting macrophages to the tumor site and VEGFR-1-positive hematopoietic progenitors from the bone marrow [32]. Moreover, PlGF can stimulate vasculogenic mimicry, chemotaxis and invasiveness of VEGFR-1-expressing cancer cells, including melanoma, further increasing their metastatic potential [17,18,33]. Nevertheless, controversial preclinical evidence is available on the antitumor activity of PlGF neutralization or gene-silencing approaches, with some studies reporting proinhibitory activity and other studies reporting protumor effects in in vivo mouse models (reviewed in [32]). Moreover, although phase 1 clinical trials with a humanized anti-PlGF mAb (TB-403 or RO5323441) showed a good safety profile [34,35,36,37], results of phase 2/3 studies have not been reported yet in the literature nor on the https://clinicaltrials.gov/ (accessed on 1 September 2022) website.

Herein, a different approach was explored to reduce the pro-tumor activity of PlGF, i.e., targeting the activation of its cognate receptor, VEGFR-1. A therapeutic advantage of VEGFR-1-specific blockade by the D16F7 mAb over PlGF neutralization is related not only to the inhibition of both PlGF- and VEGF-A-mediated signaling through VEGFR-1 but also to the ability of mAb to preserve the decoy function of the soluble receptor. In fact, sVEGFR-1, by sequestering PlGF or VEGF-A released in the ECM, reduces the amount of these angiogenic factors freely available for membrane VEGFR-1 and VEGFR-2 stimulation (reviewed in [6]). The sVEGFR-1 deposited into the ECM can also interact with α5β1 integrin expressed on the membrane of endothelial and melanoma cells, triggering their adhesion, migration and ability to settle in distant organs [22,25].

In the present study, we demonstrated that D16F7 mAb significantly hampers adhesion of endothelial or melanoma cells to sVEGFR-1, further decreasing cell motility in the ECM. In addition, even in other tumor types, such as squamous lung carcinoma, sVEGFR-1/α5β1 integrin interaction was found to be involved in disease progression and lack of response to antiangiogenic agents, including the anti-VEGF-A mAb bevacizumab [38]. Therefore, D16F7 mAb-mediated inhibition of cell adhesion to sVEGFR-1 might not only enhance the overall mAb efficacy but also improve the response to other co-administered antiangiogenic treatments. In this regard, bevacizumab was reported to cause pharmacokinetic drug interactions when associated with the anti-PlGF mAb TB-403, leading to increased plasma concentration of the latter mAb in an unbound form [39]. This has been attributed to an increase in unoccupied membrane VEGFR-1 available to bind PlGF as a result of VEGF-A depletion induced by bevacizumab and to the inability of TB-403 to neutralize PlGF when the growth factor is already bound to its receptor. Such drug interactions are unlikely to occur between the anti-PlGF and D16F7 mAbs, as the anti-VEGFR-1 mAb inhibits receptor activation but neither causes VEGF-A depletion nor affects ligand binding.

In accordance with our previous in vivo preclinical studies performed in the same syngeneic B16F10/BD2F1 melanoma model used in the present investigation [17,20], treatment with D16F7 mAb significantly inhibited melanoma growth. We found that antibody treatment markedly reduced tumor infiltration in the surrounding muscle and, more strikingly, in the bone tissue. This effect was associated with a substantial decrease in PlGF-positive melanoma cells, reinforcing the concept that either a PlGF/VEGFR-1 autocrine loop or VEGFR-1 activation by PlGF produced by other cells present in the tumor microenvironment, including tumor-associated macrophages, may favor invasion of the surrounding tissues and acquisition of a bone tropism. Our results strongly suggest that melanoma cells with an active PlGF/VEGFR-1 pathway preferentially accumulate at the bone marrow site. The antitumor activity of D16F7 mAb includes a marked inhibitory effect on the ability of melanoma to infiltrate bone marrow, reducing the levels of PlGF that can attract more VEGFR-1-expressing tumor cells. This finding is of particular relevance, given that bone is the fourth most common site of melanoma metastasis and that patients with multiple skeletal events have a dismal prognosis [40,41]. The results of in vitro studies indicated that D16F7 mAb was also able to induce a reduction in PlGF release in melanoma culture supernatants, likely contributing to a restrained tumor invasiveness.

## 5. Conclusions

**Figure 7:**
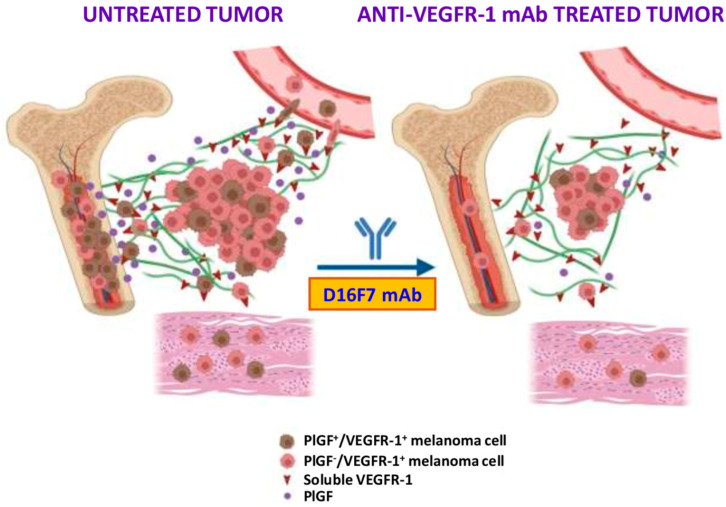
Schematic representation of the mechanisms by which PlGF/VEGFR-1 autocrine loop may favor melanoma spread and tissue infiltration. Besides the role played in M2 tumor-associated macrophages and vascular endothelium, favoring neovessel formation and tumor growth/spread, the transmembrane and soluble forms of VEGFR-1 directly modulate the ability of melanoma cells to invade the ECM, circulate into vascular vessels after crossing the endothelium layer and infiltrate tumor-surrounding tissues. In particular, the bone seems to be a preferred site for the invasion of PlGF/VEGFR-1-expressing melanoma cells. Therefore, blockade of this autocrine stimulation by the anti-VEGFR-1 D16F7 mAb hampers tumor cell spread out of the tumor mass. See text for further details. Created with BioRender.com (accessed on 24 August 2022).

Overall, our results unveil additional mechanisms underlying the antimelanoma activity of the anti-VEGFR-1 D16F7 mAb: dual blockade of membrane VEGFR-1 activation by PlGF and of integrin-mediated adhesion to sVEGFR-1, both processes that contribute to ECM invasion and tissue infiltration (Figure 7). In particular, D16F7 mAb: (a) disrupts the interaction of α5β1 integrin expressed in melanoma cells with the sVEGFR-1 deposited on the ECM by the tumor and vascular endothelium, interaction that favors cell motility through the matrix toward surrounding tissues; (b) blocks the ability of melanoma cells to enter into the bloodstream across the endothelial layer; and (c) downmodulates PlGF production. Results observed in the murine melanoma model in vivo after treatment with D16F7 mAb indicated not only a strong reduction in the tumor mass but also an almost complete elimination of bone infiltrate that, in untreated controls, presented a high percentage of PlGF-positive melanoma cells. Therefore, PlGF/VEGFR-1-expressing melanoma cells seem to possess a particular tropism toward the bone tissue, and D16F7 mAb is highly efficient in reducing tumor infiltrate at this site.

The acceptable safety profile of D16F7 mAb demonstrated in preclinical in vivo models, either as single agent or in combination with immune checkpoint inhibitors [17,20], encourages further studies aimed at mAb humanization for its future clinical evaluation in metastatic melanoma patients.

## Figures and Tables

**Table 1:** Effect of the anti-VEGFR-1 D16F7 mAb on PlGF release in the culture medium by B16F10 cells.

Experiment	Untreated Cells ^1^	D16F7-Treated Cells ^1^	% Decrease	*p* Value ^2^
1	292 ± 31	160 ± 27	45.2	<0.05
2	133 ± 27	84 ± 15	36.9	<0.05
3	271 ± 11	180 ± 11	33.6	<0.05
4	663 ± 80	400 ± 109	39.7	<0.05

^1^ PlGF released in the culture medium after 3 days of culture in the presence or absence of D16F7 mAb, expressed as pg/10^6^ cells (four experiments, each performed in quadruplicate). ^2^ *p* values for the differences between untreated and treated cells evaluated by Mann–Whitney U test.

## Data Availability

Raw data of the results presented in Figure 1, Figure 2, Figure 3, Figure 4, Figure 5 and Figure 6 and Table 1 are available in the Mendeley Data database with the following doi:10.17632/r92rysksth.1.

## Supplementary Materials

The following supporting information can be downloaded at: https://www.mdpi.com/article/10.3390/cancers14225578/s1, Figure S1: The anti-VEGFR-1 D16F7 mAb does not affect B16F10 melanoma cell proliferation; Figure S2: The anti-VEGFR-1 D16F7 mAb recognizes the murine sVEGFR-1 in immunoblot blot analysis; Figure S3: Immunofluorescence analysis of PlGF and HMB45 expression on B16F10 melanoma grafts from untreated mice.
