# Supplementary material for: The Anti-Vascular Endothelial Growth Factor Receptor 1 (VEGFR-1) D16F7 Monoclonal Antibody Inhibits Melanoma Adhesion to Soluble VEGFR-1 and Tissue Invasion in Response to Placenta Growth Factor
Source: Cancers (Basel). 2022 Nov 14;14(22):5578. doi: 10.3390/cancers14225578 (PMC9688925; doi:10.3390/cancers14225578)
Supplement: Supplementary file 1 [file cancers-14-05578-s001.zip › cancers-1969352-supplementary.pdf]

Supplementary Material

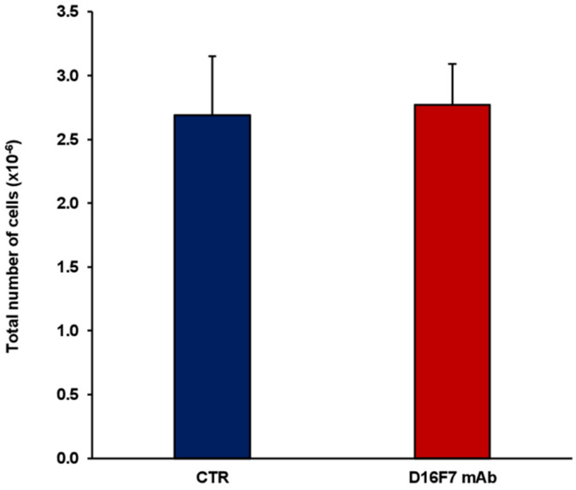

Figure S1. The anti-VEGFR-1 D16F7 mAb does not affect B16F10 melanoma cell proliferation. B16F10 cells (2.5×10^5/well in 2 mL of complete medium) were seeded in 6-well plates and allowed to grow for 48 h in the absence (control, CTR) or in the presence of D16F7 mAb (10 µg/mL). Conditioned media from B16F10 cells were obtained by incubating cultures for additional 24 h, always in the presence or absence of the antibody, in 2 mL of 0.1% BSA/RPMI 1640 medium. Cells were detached from the flasks with PBS/EDTA and counted in the presence of trypan blue to assess viability. Data are the mean ± SD of the results of four independent experiments, each one performed in sextuplicate (n = 24). Statistical analysis was performed by the Student's *t*-test (normal distribution) and the difference between the two groups were non-significant.

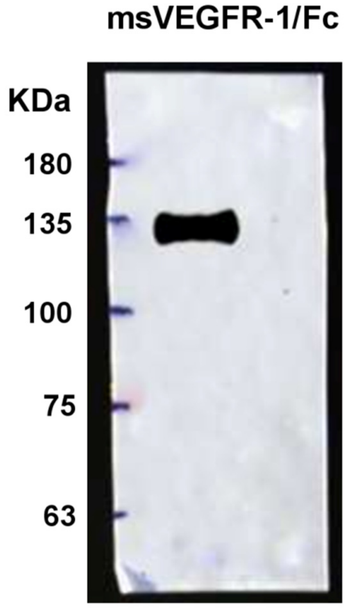

Figure S2. The anti-VEGFR-1 D16F7 mAb recognizes the murine sVEGFR-1 in immunoblot analysis. The murine VEGFR-1/Fc (1 µg) chimera was loaded on 8% polyacrylamide gels and D16F7 mAb was used for immunodetection (1 µg/mL).

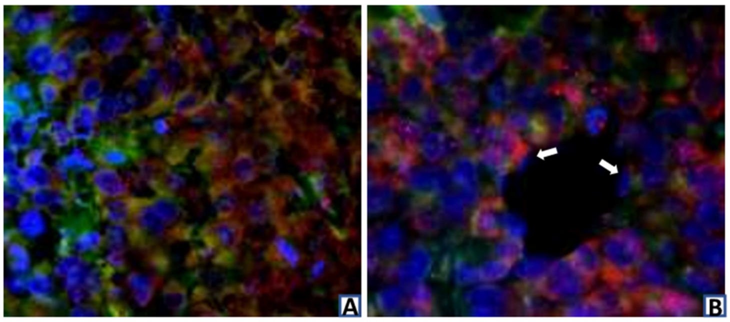

Figure S3. Immunofluorescence analysis of PlGF and HMB45 expression on B16F10 melanoma grafts from untreated mice. Data refer to the same experiment described in Figure 3D of the manuscript. (**A**) Image shows several tumor cells co-expressing PlGF (red) and HMB45 (green). (**B**) PlGF positive endothelial cells (arrows).
